# Urine miR-21-5p as a potential non-invasive biomarker for gastric cancer

**DOI:** 10.18632/oncotarget.16916

**Published:** 2017-04-07

**Authors:** Hsiao-Wei Kao, Chao-Yu Pan, Chun-Hung Lai, Chew-Wun Wu, Wen-Liang Fang, Kuo-Hung Huang, Wen-Chang Lin

**Affiliations:** ^1^ Institute of Biomedical Sciences, Academia Sinica, Taipei, Taiwan; ^2^ Institute of Biomedical Informatics, National Yang-Ming University, Taipei, Taiwan; ^3^ Department of Surgery, Veterans General Hospital and National Yang-Ming University, Taipei, Taiwan

**Keywords:** microRNA, liquid biopsy, urine samples, gastric cancer

## Abstract

Many reports have implicated that microRNAs involve in cancer development and progression, such as miR-155 in breast cancers and miR-196 in gastric cancers. Furthermore, microRNAs are more stable than typical protein-coding gene mRNAs in varieties of clinical samples including body fluids. This suggests that they are potentially valuable biomarkers for cancer monitoring. In this study, we have used urine samples of gastric cancer patients to demonstrate the feasibility of urine microRNAs for gastric cancer detection. Urine samples of gastric cancer patients were extracted for total RNA, which were examined for the expression of miR-21-5p using quantitative stem-loop PCR. Our results demonstrated that miR-21-5p could be detected in small amounts of urine samples with good stability, and the expression levels of miR-21-5p were reduced following surgical removal of gastric cancer tissues. These results implicate that urine miR-21-5p could be utilized as a novel non-invasive biomarker of gastric cancer detection and monitoring.

## INTRODUCTION

Gastric cancer occurs frequently worldwide and remains prevalent despite the declining incidence in recent decades [1]. It is the second leading cause of global cancer death and a malignant disease with high mortality rate in Taiwan [2, 3]. Unfortunately, early gastric cancer diagnosis is not feasible for most gastric cancer patients due to the lack of useful and convenient non-invasive detection biomarkers [4]. It is essential to explore clinical non-invasive biomarkers for gastric cancer detection and monitoring.

microRNAs (miRNAs) are short non-coding RNAs of 22 nucleotides, which are transcribed by RNA polymerase II and interacting with 3′ untranslated region of target genes [5]. Thus, miRNAs involve in gene expression regulation at post-transcriptional level. There are over 2,500 miRNAs identified in human [6]. Owing to involvements in transcriptional and post-transcriptional regulation of multiple target genes, the overall miRNA modulation networks are quite complicated. Some miRNAs could act as tumor-suppressors, and others could serve as oncogenic miRNAs (oncomiRs). Many reports demonstrated the significance of miRNAs in human cancers [7–9]. Our laboratory has previously implicated the importance of several miRNAs in gastric cancers, including miR-21, miR-34, miR-129 and miR-196 [10–12]. It is thrilling to found that miRNAs can be released from cancer cells into body fluids via secreting exosomes particles [13, 14], which would protect them from degradation in circulation. So far, they have been detected in human serum, plasma, urine, saliva, tears and amniotic fluid etc [15]. Therefore, circulating miRNAs could be utilized as novel liquid biopsy biomarkers.

For routine clinical cancer diagnoses, needle biopsy, endoscopy, imaging methods together with blood test are commonly used techniques. New advanced techniques for non-invasive diagnosis of gastric cancers, such as surface-enhanced Raman scattering sensors used in detecting volatile breathe organic compounds in patients, are also reported [16]. In recent years, liquid biopsy based molecular profiling and cancer monitoring have been extensively studied using circulating tumor cells and cell-free nucleic acids isolated from bloodstream [17–19], including miRNAs [20]. Mitchell et al demonstrated that expression levels of circulating miRNAs in serum are consistent with tumor tissues, and could serve as a biomarker for cancer detection [21]. Plasma miR-16-5p and miR-19b-3p were shown to be potential biomarkers for gastric cancer progression by Zhang et al [22]. Our laboratory has also demonstrated the significant association of serum miR-196s in the recurrence of gastric cancers [11]. However, blood collection is still relatively invasive and often requires the assistance of medical professionals. Therefore, urine seems to be a better alternative source for liquid biopsy in cancer diagnosis and routine cancer monitoring [23, 24]. For example, utilization of compositions of urine samples, which has been commonly used to diagnose various human disorders, such as glucose in diabetes, ketones in ketonuria and protein concentrations in kidney disorders. The urine collection process is truly non-invasive and it is relatively safe and convenient comparing to blood drawing. Therefore, urine miRNAs could be novel clinical cancer biomarkers due to their extended stability in urine as well as their significance in cancer cells [24]. However, there is no report regarding the urine miRNAs detection from gastric cancer patients. In this article, we measure urine miR-21-5p from gastric cancer patients, intending to demonstrate the feasibility and utility of specific urine miRNAs as diagnostic and prognostic gastric cancer biomarkers.

## RESULTS

Since there were only few reports on the miRNA expression from urine samples [25–27], we have first investigated the stability of urinary miRNAs. As reported by others, circulating miRNAs are quite stable in body fluids, including urine samples. We found that U6 small nuclear RNA (snRNA) and miR-182-5p level were fairly constant in fresh collected urine samples from healthy donors (Supplementary Figure 1). It demonstrated the stability of urine miRNAs and the potential of collecting urine samples for routine biomarker detections. We also investigated the benefits of cation chelating agents (such as EDTA) in the preservation of urine miRNAs. Actually, there is no significant beneficial effect for miRNA detection by including EDTA in the urine collection vessels in our study (not shown). Nonetheless, cell-free genomic DNA in the urine samples did show less degradation with addition of EDTA as reported previously [28].

**Figure 1:**
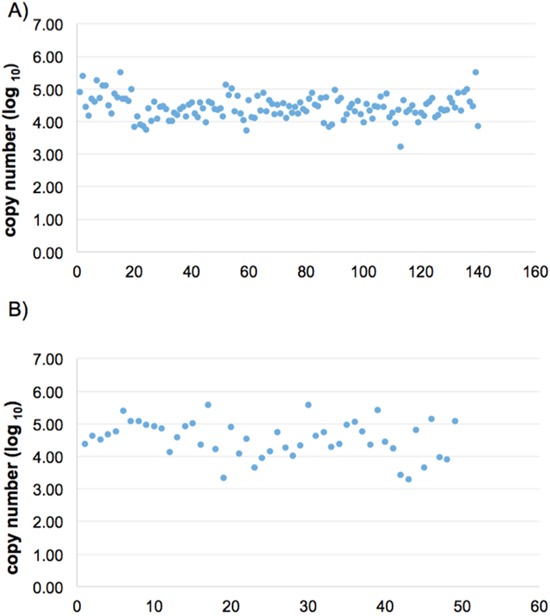
Expression levels of U6 snRNA in urine samples of healthy individuals and gastric cancer patients We measured the U6 snRNA expression levels of healthy donor urine group **(A)**, n=124 and gastric cancer patient group **(B)**, n=49. Each dot represents an individual urine sample.

We measured the relative expression level of urine miRNAs by comparing with U6 snRNA level. U6 snRNA is used a common reference standard in miRNA expression studies [29]. We have further examined the urinary U6 expression using urine samples collected from large-scale cohort community studies (The Taiwan BioBank Project), where donor samples came from various collection stations. We have examined 140 individual samples from BioBanK collection, and obtained the expression of U6 on 124 urine samples by the TagMan miRNA protocol with good success (Figure 1A). Similar expression level of urine U6 from gastric cancer patients was observed (Figure 1B).

Previously, our laboratory has demonstrated that miRNAs could be readily detected in the clinical samples of gastric cancer patients, including tissues and serum [10, 12, 30, 31]. We have demonstrated the increased expression of several miRNAs in gastric cancer tissues and significant association of serum miR-196s in the recurrence of gastric cancer [11]. Recently, we have further generated NGS reads from small RNA libraries of several gastric cancer tissues, and identified differential expressed miRNAs in gastric cancer [32]. About twenty up-regulated oncogenic miRNAs were discovered, including some of the known oncomiRs, such as miR-21-5p. miR-21-5p is a well-known highly expressed oncomiR, and its expression has been reported to be elevated in several cancer types including gastric cancer [7]. Serum miR-21 expression has been shown to be an effective biomarker for different human cancers using a meta-regression analysis on thousands cancer patients [33]. Therefore, we would like to interrogate the expression levels of miR-21-5p in the urine samples of gastric cancer patients in this study. In particular, we would like to investigate the association of urinary miR-21-5p level following surgical removal of gastric cancer tissues.

**Figure 2:**
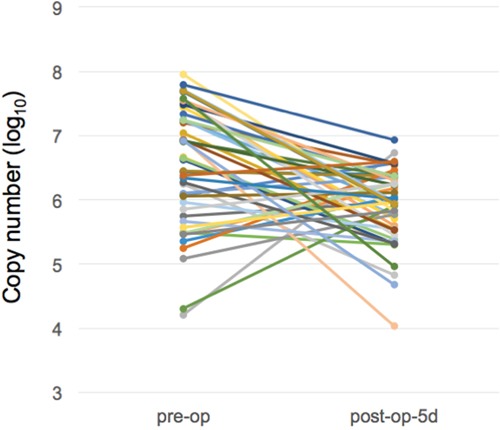
Expression levels of miR-21-5p in urine samples of gastric cancer patients before and after surgery We measured the miR-21-5p expression levels between pre-operation group and post-operation group (5 days following surgery) (n=48).

**Figure 3:**
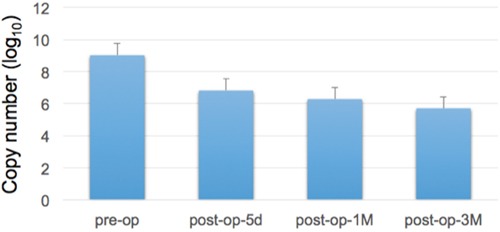
Expression levels of urine miR-21-5p at different time points following surgery We measured the miR-21-5p expression levels before and after surgery in a represented gastric cancer patient (5 days following surgery, one Month following surgery, and three Months following surgery). Reduced miR-21-5p expression level was observed following the removal of cancer tissues.

**Figure 4:**
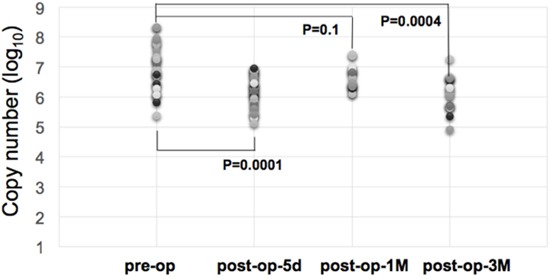
Expression levels of miR-21-5p in urine samples of gastric cancer patients following surgery Temporal distribution of urine miR-21-5p expression levels in different groups of gastric cancer patients following surgery. Before surgery (n=26), 5 days following surgery (n=34), one Month following surgery (n=16), and three months following surgery (n=17). Statistical significance was then determined between the pre-op group and post-op groups by Student's t-test.

As shown in Figure 2, we demonstrated that there were differences in miR-21-5p expressions between urine samples collected before and after surgical operations of gastric cancer patients. Comparing with the miRNA expression levels in gastric cancer urine samples from pre-operation and post-operation groups, the expression levels of miR-21-5p showed a significant decrease trend after the removal of cancer tissues (p-value = 0.00022, Mann-Whiteny U test). This suggests that miR-21-5p could be utilized as the non-invasive candidate biomarker for detecting gastric cancers. This data also well reflects the previous observation on the significant miR-21-5p expression pattern in gastric cancers. As illustrated in Figure 3, with a single individual patient, it is clearly demonstrated that urine miR-21-5p is greatly reduced following surgery, and the level of miR-21-5p continued to be decreased at 1 Month and 3 Months after surgery. Similar reduction patterns could be observed in additional numbers of patients (Figure 4).

**Figure 5:**
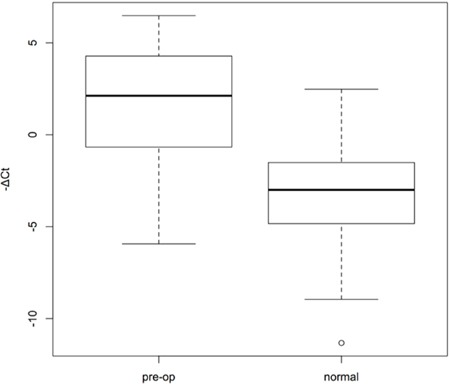
Box plots of urine miR-21-5p expression levels of gastric cancer patients and healthy individuals We measured the urinary miR-21-5p expression levels of gastric cancer patients as well as healthy donors. -ΔCt means for relative expression of miRNAs, which was calculated by following equation: -(Ct value of miR-21-5p minus Ct value of U6RNA). The statistical difference between the two groups was determined with Mann-Whitney U test with the p-value of 3.532e-15.

Thus, miR-21-5p could be used as a potential non-invasive biomarker for monitoring the status of gastric cancer disease status. Using the BioBank samples as baseline, we found that urine miR-21-5p level is significantly up-regulated in gastric cancer patients comparing with healthy individuals (Figure 5). The statistical p-value is 3.532e-15 with Mann-Whiteny U test. Our data implicated that urine miR-21-5p indeed could serve as a useful non-invasive biomarker for monitoring gastric cancer status.

## DISCUSSION

Owing to the complicated oncogenesis mechanisms and therapeutic difficulties of gastric cancer, it is necessary to develop more sensitive and accurate method for early diagnosis as well as prognosis. Even though there are some medical examination procedures, such as needle biopsy and endoscopy can provide accurate diagnosis results, researchers still make every effort to lower the cost and develop non-invasive tests for reduce patient's burden. Checking urinary miRNA expression level is a desired way for being a non-invasive test and for routine cancer monitoring [27]. We demonstrate that some miRNAs are stable in urine samples for an extended period of time, even under routine urine collection conditions in outpatient clinics. This implicated that urine miRNAs could be a new source for non-invasive cancer monitoring. There are many previous reports of urine miRNAs as biomarkers for urological cancers (bladder cancer, prostate cancer) [25, 34–40]. These publications have shown that miRNAs can enter circulating system by secretion via exosomes pathways from cancer cells or release from apoptotic or necrotic cancer cells [41, 42]. This demonstrated the utilities of urine miRNAs for cancer biomarkers. However, these reports of urine miRNAs were mostly investigating the excretory or genitourinary cancer system tumors. Our report here demonstrates the applications of urinary miRNAs for solids tumors of internal organs, which is a significant finding for the identification of non-invasive cancer biomarkers of human cancers from internal organs. Besides our findings, it is recently demonstrated urinary miRNAs could be used for breast cancer detection. miR-155 was found to be elevated in the urine samples of breast cancer patients [43]. Three miRNAs (miR-223, miR-142 and miR-30e) were found to be elevated in the urine samples of pancreatic ductal adenocarcinoma patents [44]. Therefore, it is possible that urinary miRNAs could be used for other solid tumors.

Liquid biopsy is now a widely studied area using circulating tumor cells and cell-free DNA or miRNA in the blood stream as diagnosis and prognosis biomarkers [17]. Many studies have reported the utilization of serum miRNA detection in various human cancers as biomarkers [21, 45–52]. However, cell-free DNA/RNA in the urine has been rather neglected for clinical cancer research applications comparing to the blood based biomarker studies. From our results, it is feasible to use urinary miRNAs as a routine liquid biopsy platform for solid tumors. Current stem-loop RT-PCR for miRNA detection is widely adapted for the measurement of specific miRNA population, which is fairly reliable and reproducible. Using synthetic miRNA sequence oligos as quality control benchmarks, we found the TagMan miRNA measurement platform is reliable and reproducible in different time intervals. Nevertheless, the starting molecules in the reaction limit PCR assay performance and urine samples do have the volume dilution issues. Therefore, use of U6 snRNA or other high abundant snRNAs might be a good indication of urine quality.

In conclusion, our data shows that miR-21-5p is highly expressed in urine samples of gastric cancer patients with tumor burdens. We have demonstrated that the expression level of miR-21-5p in the same patient was reduced after surgical resection of gastric cancer tissues. This implicated the potential of urine miRNAs as valuable non-invasive cancer monitoring biomarkers in the future liquid biopsy applications.

## MATERIALS AND METHODS

### Clinical urine samples

Urine samples of gastric cancer patients were obtained from patients who had undergone gastric resection at the Department of Surgery, Veterans General Hospital-Taipei, Taiwan [53]. The study was approved by the Institutional Review Board, and informed consent was obtained from all patients before surgery. All urine samples were collected from spontaneous voids before surgery and post-surgery. In total, around 50 gastric cancer patients were recruited for urine samples collection. The urine samples were collected at different time point before surgery and during outpatient follow-up: pre-op (pre-operation), post-op (post-operation) 5 days, post-op 1 month and post-op 3 month. 35 to 45ml urine samples were collected using 50ml corning tube mixing with 5ml 0.5M EDTA (pH 8.0). Urine samples were kept at 4°C at first and then stored in −20°C for long-term storage. Control delinked urine samples were obtained from healthy volunteers recruited in the Taiwan BioBank cohort study.

### Total RNA extraction

Urine total RNA was extracted from 250 μl urine samples using 2.5-fold volume of TRIZOL reagent (Invitrogen; Carlsbad, CA, USA), mixed thoroughly by vortexing for 30 seconds and incubated on ice for 5 min. 150 μl of chloroform was then added to each aliquot, then vortexed for 1 min followed by centrifuging for 5 min at 12000 rpm. The supernatant was retained, extracting with equal volume of TRIZOL and repeating the above-mentioned procedure. The resulting supernatant was precipitated by ETOH overnight at −20°C. Finally, total RNA was resuspended in 40 μl of RNase-free H_2_O. In general, we obtained 30 to 40 ng total RNA was obtained from 250 μl urine samples.

### Quantitative stem-loop RT-PCR with TagMan probes

4.5μl of total RNA (around 4 ng total RNA) was reverse-transcribed with miRNA-specific stem-loop RT primers using SuperScript III reverse transcriptase in 10 μl volume (Invitrogen; Carlsbad, CA, USA). 3μl RT products were then used to perform quantitative stem-loop RT-PCR reaction for miRNA detection with ABI Step-One Plus. For each reaction, RT product, 1μl specific primer (2μM) and 0.5μl universal primer (2μM) were added with 0.5μl TaqMan probe (2.5μM) in a 10μl reaction volume. The reaction was performed with the following incubation conditions: 30 min at 16°C, followed by (20°C for 30s, 42°C for 30 s, 50°C for 1 s) for 50 cycles. Relative quantification of miR-21-5p was calculated by the ΔCt method normalized on corresponding expression level of U6 snRNA, and copy numbers of expression is then calculated with synthetic oligo standards. The specific PCR primer pairs are listed here:

Universal primer:

5′-CAACTGGTGTCGTGGAGTCGG

U6 forward primer:

5′-CTCGCTTCGGCAGCACATATACT

U6 reverse primer:

5′-ACGCTTCACGAATTTGCGTGTC

miR-21-5p (RT primer):

5′-CTCAACTGGTGTCGTGGAGTCGGCAATTCAGTTGAGTCAACATC

miR-21-5p (Q-PCR primer):

5′-CGGCGGTAGCTTATCAGACTGA

miR-182-5p (RT primer):

5′-CTCAACTGGTGTCGTGGAGTCGGCAATTCAGTTGAGAGTGTGAG

miR-182-5p (Q-PCR primer):

5′-CGGCGGTTTGGCAATGGTAGAA

### Statistical analysis

The statistical analyses were performed by using the open source statistical software environment R. Mann-Whitney U test was applied to examine the significance of median expression levels of urinary miR-21-5p.

## SUPPLEMENTARY MATERIALS FIGURES AND TABLES


