# Supplementary material for: Urine miR-21-5p as a potential non-invasive biomarker for gastric cancer
Source: Oncotarget. 2017 Apr 7;8(34):56389–97. doi: 10.18632/oncotarget.16916 (PMC5593569; doi:10.18632/oncotarget.16916)
Supplement: Supplementary file 1 [file oncotarget-08-56389-s001.pdf]

Urine miR-21-5p as a potential non-invasive biomarker for gastric cancer

SUPPLEMENTARY FIGURE

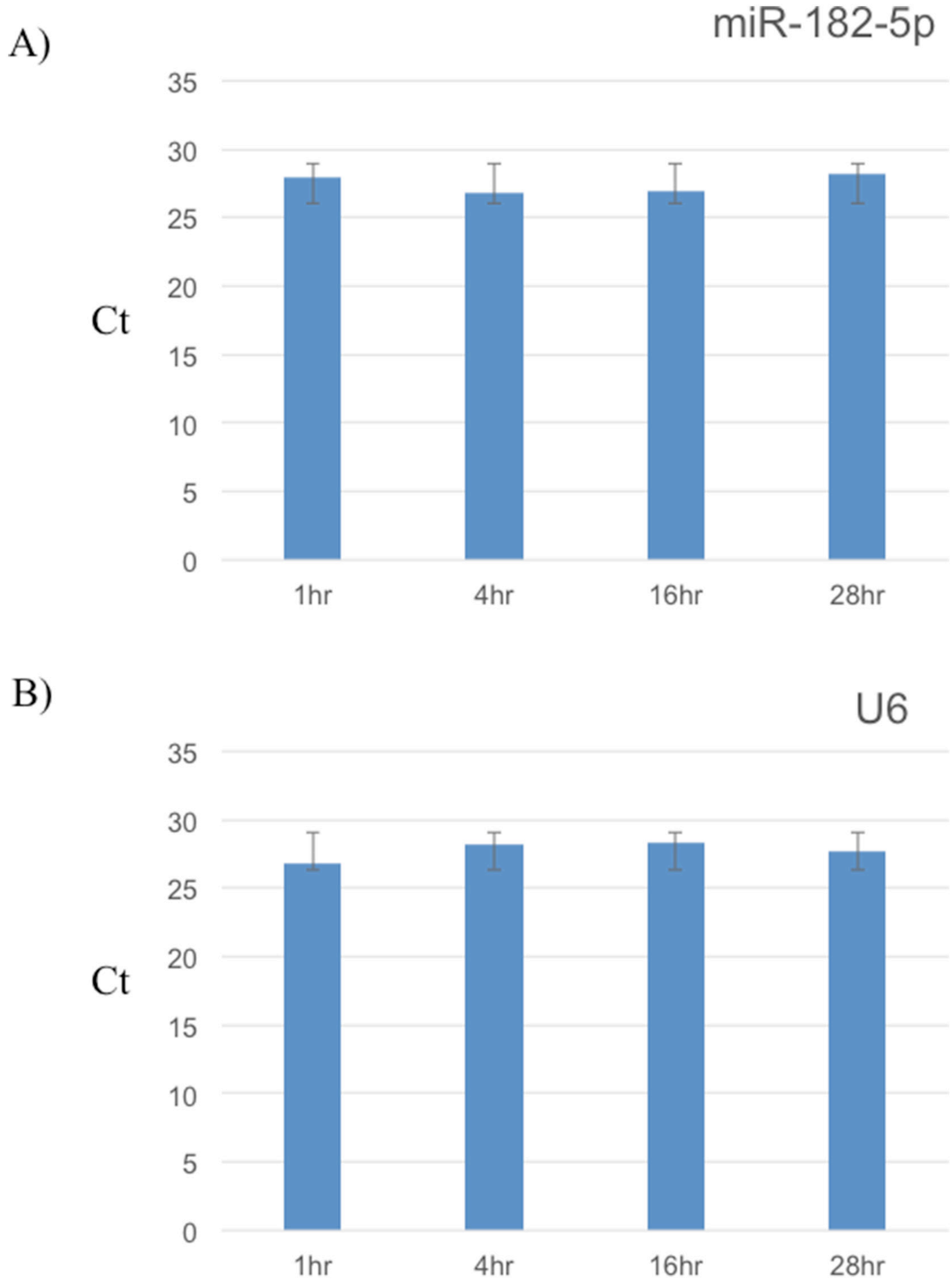

Supplementary Figure 1: miRNA stability during collection phase. miRNA and small nuclear RNA expression were examined using healthy donor urine samples collected from different storage time periods. **(A)** Expression of miR-182-5p was examined at 1 hour, 4 hours, 16 hours and 28 hours storage at room temperature. **(B)** Expression of U6 snRNA was examined at 1 hour, 4 hours, 16 hours and 28 hours storage at room temperature. The miRNA expression was measured with triplicate experiments. miR-182-5p was selected due to its relatively high expression level in our previous miRNA-Seq experiment [28].
